# The multi-agency partnership roadmap for newborns in humanitarian settings: Timely and crucial during the COVID-19 pandemic

**DOI:** 10.7189/jogh.11.03015

**Published:** 2021-01-16

**Authors:** Saverio Bellizzi, Gabriele Farina, Maura Fiamma, Giuseppe Pichierri, Paola Salaris, Catello M Panu Napodano

**Affiliations:** 1 Medical Epidemiologist, Independent Consultant, Geneva, Switzerland; 2 University of Sassari, Sassari, Italy; 3 Ospedale San Francesco, Nuoro, Italy; 4 Kingston Hospital NHS Foundation Trust, Microbiology Unit, Kingston Upon Thames, UK; 5 Mater Olbia Hospital, Olbia, Italy

Despite the fact that mortality rates for COVID-19 seem to be low in children and in women of reproductive age, these groups might be disproportionately affected by the disruption of routine health services in low- and middle-income countries, especially in fragile and humanitarian settings.

A very recent study traced all babies less than 29 days old with COVID-19 across the UK and confirmed that severe infection in newborn babies is still very rare. While the main symptoms of the infection included high temperature, poor feeding, vomiting, cough and lethargy, the study found that 1 in 1785 newborns (0.06% of births) required hospital treatment and a small proportion of babies caught COVID-19 from their mother (only 17 out of 66 newborns were suspected to have caught the virus from their mother in the first seven days of life). This reinforces the concept whereby a baby does not need to be separated from his mother if she tests positive for COVID-19 [1].

Countries facing conflict and political instability have the highest rates of neonatal mortality and stillbirths: if India and China are excluded, countries experiencing chronic conflict or political instability account for approximately 42% of all neonatal deaths worldwide [2].

While it is important to point out that even in the most precarious situations, many of the deaths that occur around the time of birth are preventable [3], a recent report showed how reduced coverage of antibiotics for pneumonia, neonatal sepsis and diminished access to rehydration solution for diarrhea would together account for around 41% of additional child deaths during the COVID-19 pandemic [4].

As correctly underscored by Brenda Sequeira Dmello and colleagues [5], the consequences on maternal and newborn health of disruption of health services in the context of fragile health care systems could be devastating and one of the critical aspect is the complex procedure of contextualizing recommendations in such settings due to scarcity of data [6].

Humanitarian crises threaten the health and safety of communities directly and through the destruction of existing health systems and infrastructure, with pregnant women and newborn especially vulnerable. Based on these needs, a Declaration to Accelerate Newborn Health in Humanitarian Settings was released in February 2019 by key stakeholders from multiple sectors within the humanitarian and development fields, co-convened by Children, UNICEF, UNHCR and WHO, to catalyze a global agenda for improving newborn health in humanitarian settings. This resulting declaration was a call for the dignity, health, and well-being of every woman, every child, and every newborn – in humanitarian and fragile settings – to be urgently upheld and prioritized [7].

**Figure:**
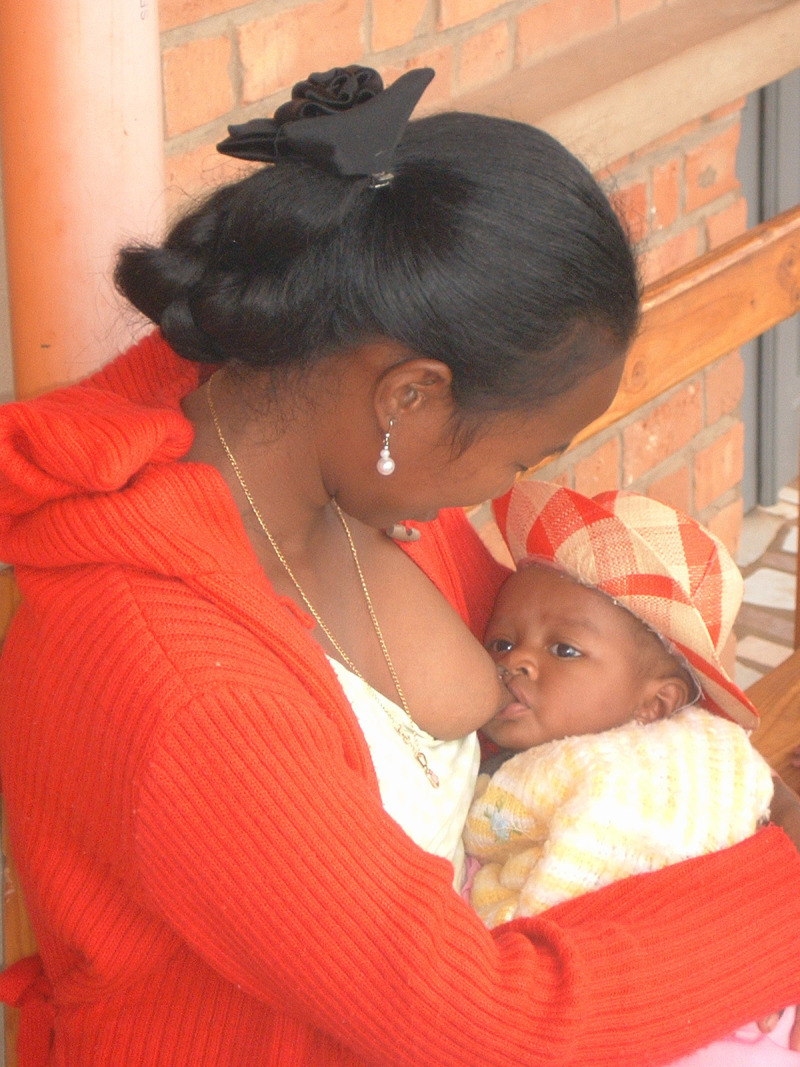
Photo: This picture shows a mother breastfeeding her baby in a rural community in Mozambique (source: private photo repository, Saverio Bellizzi, used with permission).

The close collaborative effort triggered the production of a dedicated strategy for newborn health in humanitarian and fragile settings with the contribution and benefit of inputs of a wide range of stakeholders, including clinicians, implementers, academics, policymakers, government representatives, donors, private sector representatives, and professional associations across the reproductive, maternal, neonatal, child and adolescent health and nutrition continuum.

The Roadmap to accelerate progress for every newborn in fragile and humanitarian settings 2020-2025 calls for collective and accountable action. It emphasizes the need to engage stakeholders from across humanitarian and development sectors to ensure that mothers and newborns – the essential dyad – survive and thrive even in the most difficult circumstances and across all phases of emergency response using a health systems approach.

The roadmap clearly emphasizes the fact that the mother-newborn relationship is often underestimated or overlooked, and interventions such as promoting early and exclusive breastfeeding and skin-to-skin care are at risk of being de-prioritised during humanitarian response, despite their cost-effectiveness. In this regards, promotion and support for early initiation and exclusive breastfeeding are lifesaving interventions that should be provided during humanitarian response for both healthy and high-risk newborns [8].

It is particularly important that staff receive training on neonatal resuscitation to address asphyxia, and that they implement kangaroo mother care (KMC), feeding support, and monitored oxygen for premature babies [9].

Leadership by national and local governments is fundamental to contribute to rapid improvements in maternal and newborn survival during crises. This leadership is vital towards maintaining sustainable progress. Governments can develop policies and allocate resources to ensure mothers, pregnant women and newborns receive the care they need during an emergency. Health system resilience at national and sub-national levels should be strengthened by integrating priority maternal and newborn health interventions into preparedness and response plans, using global guidance and evidence to inform policies.

The Roadmap to accelerate progress for every newborn in fragile and humanitarian settings 2020-2025 represents an important momentum for the existing workstream on newborn health in humanitarian settings and is even more relevant during the current pandemic to raise the voices of children and mothers in the highest mortality and morbidity burden zones of the world.
